# Isolation of theca cells from human antral follicle membranes using ANPEP-based magnetic cell sorting

**DOI:** 10.1007/s10815-026-03983-2

**Published:** 2026-07-23

**Authors:** Lea Bejstrup Jensen, Malene Louise Johannsen, Jane Alrø Bøtkjær, Anna Ronnefeld Veng, Kirsten Tryde Macklon, Anette Tønnes Pedersen, Bjarne Styrishave, Stine Gry Kristensen

**Affiliations:** 1 Laboratory of Reproductive Biology, University Hospital of Copenhagen, Rigshospitalet, Copenhagen 2100, Denmark; 2 Drug Toxicology, Metabolism and Analysis Group, Department of Pharmacy, University of Copenhagen, Copenhagen 2100, Denmark; 3 The Fertility Clinic, Department of Gynaecology, Fertility and Obstetrics, University Hospital of Copenhagen, Rigshospitalet, Copenhagen 2100, Denmark; 4 Department of Clinical Medicine, University of Copenhagen, Copenhagen 2200, Denmark

**Keywords:** Theca cells, Granulosa cells, ANPEP, Magnetic-activated cell sorting (MACS), Flow cytometry, Steroidogenesis

## Abstract

**Research question:**

Can ANPEP-based magnetic activated cell sorting (MACS) be used to isolate and functionally enrich human steroidogenic theca interna cells from small antral follicle membranes?

**Methods:**

This experimental study used ovarian tissue from eight women (mean age 33.7 years) undergoing fertility preservation. Membranes from small antral follicles (2–9 mm) were enzymatically digested and cultured for 48 h. A mixed cell sample was obtained before MACS separation into ANPEP-positive and ANPEP-negative cell fractions, followed by an additional 48-h culture. Cell purity and viability were assessed by flow cytometry (FACS) using ANPEP and FSHR antibodies. Gene expression (*ANPEP, CYP17A1, LHCGR, FSHR, CYP19A1*) was analyzed by RT-qPCR. Steroid secretion (pregnenolone, progesterone, 17-OH-pregnenolone, 17-OH-progesterone, DHEA, androstenedione) was quantified using LC–MS/MS.

**Results:**

Before cell sorting, 29.6–82.9% of viable cells expressed ANPEP. After MACS, ~ 68% of cells in the ANPEP-positive fraction expressed ANPEP, compared with ~ 3% in the negative fraction (*P* < 0.05). *CYP17A1* expression was higher in pre-MACS and ANPEP-positive populations than in the negative fraction. ANPEP-positive cells secreted androgen precursors (17-OH-pregnenolone, 17-OH-progesterone, DHEA, androstenedione), whereas the negative fraction produced relatively more progesterone, consistent with theca cell enrichment.

**Conclusions:**

ANPEP-based MACS enables enrichment of viable, steroidogenic human theca interna cells. The ANPEP-positive fraction exhibits molecular and functional characteristics consistent with theca lineage and provides a useful model for studying ovarian steroidogenesis.

**Supplementary Information:**

The online version contains supplementary material available at https://doi.org/10.1007/s10815-026-03983-2.

## Introduction

Theca cells are integral components of ovarian follicles from the secondary stage onward, providing structural support and driving steroidogenesis. Notably, cells of the theca interna synthesize androgens, making them essential for follicular development and maturation. This central role underscores the importance of theca cells in the development of advanced in vitro models and in elucidating the pathophysiology of theca cell-associated disorders. To better understand the functional role of theca cells, it is important to consider their structural organization within the follicle. The theca tissue section is composed of two distinct cell layers: the theca interna and the theca externa [1–3]. These layers are located outside the basement membrane, which separates them from the oocyte and the surrounding cumulus and mural granulosa cells. The theca interna comprises the endocrine cells, producing progestins and androgens, whereas the theca externa consists of fibroblast-like cells that form a supportive connective tissue layer [2]. Notably, the theca tissue is highly vascularized, facilitating efficient delivery of gonadotropins to the follicle [4].

The biosynthesis of estradiol involves a series of tightly regulated enzymatic steps, beginning with cholesterol, the fundamental building block for all steroid hormones [5–7]. Cholesterol is transported from the outer to the inner mitochondrial membrane through the action of the Steroidogenic Acute Regulatory protein (StAR) [8, 9], where Cytochrome P450 Family 11 Subfamily A Member type 1 cleavage enzyme (CYP11A1) catalyses the rate-limiting conversion of cholesterol into pregnenolone [10]. Pregnenolone can further be converted into progesterone, which is considered a key intermediate and a major downstream product in steroid hormone biosynthesis, in both theca and granulosa cells.

A defining feature of ovarian steroidogenesis is the spatial separation of the two enzymes CYP17A1 and CYP19A1 [5, 11, 12]. CYP17A1, expressed exclusively in theca cells, possesses both 17α-hydroxylase and 17,20-lyase activities, enabling the conversion of pregnenolone to dehydroepiandrosterone (DHEA) via 17-OH-pregnenolone. DHEA is subsequently converted to androstenedione, a minor fraction of which is further converted to testosterone. The majority of androstenedione diffuses across the basement membrane into granulosa cells, where it is primarily aromatized into estrone by aromatase (CYP19A1). Estrone will subsequently be reduced to estradiol by 17 β-hydroxysteroid dehydrogenase type 1 (17 β HSD1) [13]. Follicle-stimulating hormone (FSH) binds the FSH receptor (FSHR) expressed on the granulosa cells, activates cAMP-dependent signalling, upregulates CYP19A1 and 17 β HSD1 expression, and thereby drives estradiol synthesis [14–16].

Studies have shown immunofluorescent localization of aminopeptidase-N (ANPEP/CD13), a membrane-bound metalloprotease, on the surface of theca interna cells in secondary follicles, luteinized thecal cells, and the small luteal cells within preovulatory follicles and the corpus luteum [3, 17]. Importantly, ANPEP was absent in granulosa cells, indicating that ANPEP could be a theca cell throughout follicular development [3]. Furthermore, a study investigating the different subtypes of theca cells observes co-expression of ANPEP and CYP17A1 in the theca interna cells adjacent to the mural granulosa cells [18].

Despite their central role, access to primary human theca cells remains limited compared to granulosa cells, which are routinely obtained during assisted reproduction. In contrast, theca cells must typically be isolated from follicular membranes within ovarian tissue, typically sourced from organ donors, fertility preservation procedures, or through in vitro differentiation of stromal cells [17, 19–21]. Existing isolation methods rely largely on enzymatic digestion combined with flow cytometry, which can be technically demanding and may affect cell integrity. Magnetic-activated cell sorting (MACS) represents a potentially less disruptive alternative, with advantages in technical accessibility and in preserving cell viability [22–24].

Addressing this gap, the present study aimed to assess whether ANPEP-based MACS can reliably enrich theca interna cells from membranes collected from small antral follicles in the human ovary. Follicular membranes were obtained from the ovaries of young women undergoing fertility preservation during the natural cycle, enabling the establishment of a primary theca cell population from unstimulated ovaries. ANPEP-based MACS was utilized to separate theca cells from stromal and granulosa cell populations, followed by validation of ANPEP-positive cells representative of the steroidogenic theca interna lineage. The reliability of the isolation technique and the functionality of the isolated cells were assessed through flow cytometric analysis of cell viability and marker expression, gene expression profiling, and quantification of steroid hormone secretion using liquid chromatography tandem-mass spectrometry (LC–MS/MS).

## Materials and methods

### Ethics

The study was conducted in accordance with the guidelines of the Declaration of Helsinki and was approved by the Scientific Ethical Committee for the Capital Region of Denmark (J. no.: H-2–2011–044). The storage and collection of patient data were approved by the Ministry of Health (J. no. 30–1372) and by the Danish authorities to comply with the European Union. Furthermore, written and oral informed consent were obtained from all participants before their inclusion in the study.

### Patients and collection of follicle membranes

**Fig. 1:**
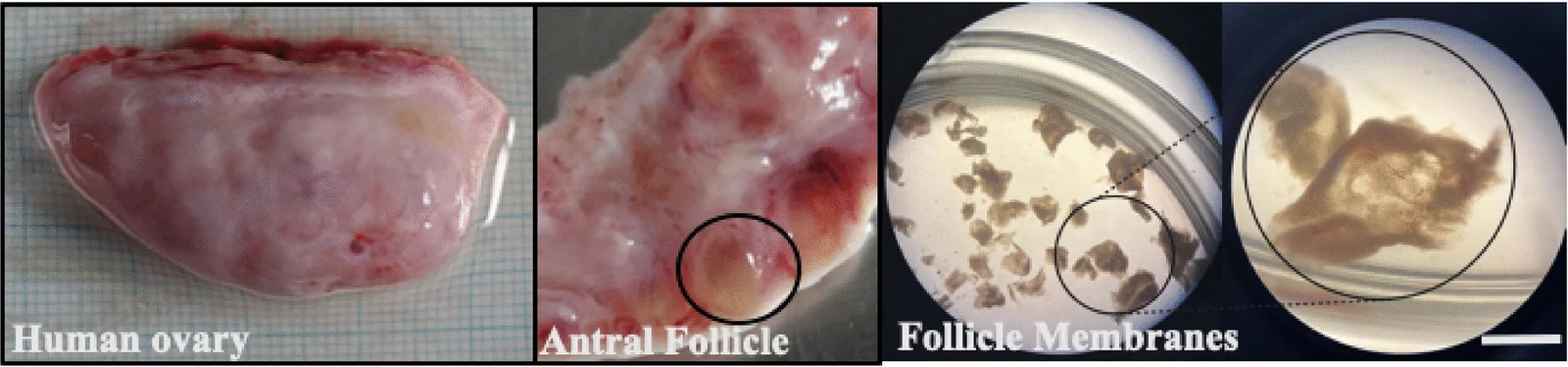
Whole adult human ovary following oophorectomy. The ovary was bisected to expose internal structures. The black circle indicates a small antral follicle located within the medulla. Follicular membranes from small antral follicles were isolated using forceps. A vascularized follicular membrane is shown. Scale bar = 2 mm. Images from the Laboratory of Reproductive Biology, University Hospital of Copenhagen, Denmark

Follicle membranes were retrieved from small antral follicles following aspiration of follicular fluid. After sectioning the ovary into two halves to facilitate access to the follicles, the membranes were carefully peeled from the inner follicular wall using forceps; while some could be removed intact, others required gradual dissection into smaller pieces, as shown in Fig. 1.

Ovarian material was donated by patients undergoing ovarian tissue cryopreservation (OTC) for fertility preservation at the University Hospital of Copenhagen, Denmark. Eight patients donated ovarian material for research. The average age of the patients was 33.7 years (range 27 to 35 years of age). They were all diagnosed with breast cancer and did not receive gonadotoxic cancer treatment before ovariectomy for cryopreservation.

### Chemicals and reagents

The culture media contained α-MEM media (Gibco™ Life Tech. 22,571–020), 1% Glutamax + (Gibco™, Life Tech. 35,050–08), 0.01% Insulin-Transferrin-Selenium (ITS) 100 × (ThermoFisher 41,400,045), 5% Human Serum Albumin (HSA) (CSL Behring, 109,697, 20%), 1% Penicillin/Streptomycin (Invitrogen 15,070–063), and 1% Human Platelet Lysate (StemCell Technologies, 06960). The holding media comprised McCoy’s 5 α culture medium containing 25 mM HEPES (Invitrogen, Gibco 22,330–21), supplemented with 1% Glutamax +, 0.01% ITS, 5% HSA, and 1% Penicillin/Streptomycin (Invitrogen 15,070–063). The digestion media comprised holding media supplemented with 0.2 mg/ml collagenase IV (Sigma-Aldrich, St Louis, MO, USA) and 0.2 mg/mL DNase I (Sigma-Aldrich, St Louis, MO, USA). The media for terminating the digestion contained holding media supplemented with 10% Fetal Bovine Serum (Invitrogen, Gibco 10,500–064). Separation buffer: 0.5% BSA in 1 × PBS + 2 mM EDTA (1.5 g BSA in 300 mL PBS + 0.0223 g EDTA, sterile filter (0.2 µm).

For washing of the cells, phosphate-buffered saline (PBS) (Gibco™, Life Tech., 1 ×) and HEPES buffer solution (Gibco, Cat: 15,630,049) were used. Plating of cells: Fibronectin (Sigma Aldrich, F0895), Hepes BSS solution, Accumax (Sigma-Aldrich, A7089).

For RNA isolation: Trizol reagents (Ambion Life Technologies, 15,596,026), 1-bromo-3-chloropropane (Sigma, B9673-200ML), Ethanol 99%, DEPC H_2_O, and RNeasy minikit 250 (Qiagen, 74,106). RNA isolation of cDNA synthesis was performed using the high-capacity cDNA reverse Transcription Kit (Applied Biosystems™, 4,368,814). Magnetic activated cell sorting, anti-Biotin MicroBeads (Miltenyi biotec, 130–090–485), biotin Anti-ANPEP antibody WM15 (abcam, ab130430), MS Columns (Miltenyi Biotec, 130–042–201), separation media, culture media, MACS multistand, MiniMACS separator. Flow cytometry, 2% Paraformaldehyde (PFA), 10% goat serum, PE anti-human ANPEP Ab (BioLegend, 301,704), VIACOMP® (SLiNGSHOT, SSB-07-A), SPECTRACOMP® XT (SLiNGSHOT, SSB-21-A), Viability stain (BD, 562,247), APC anti-human FSHR Ab (Biotechne, FAB65591A), Corning® Costar® Ultra-Low Attachment Multiple Well Plate (Merck, CLS7007).

### Enzymatic digestion and magnetic activated cell sorting (MACS)

**Fig. 2:**
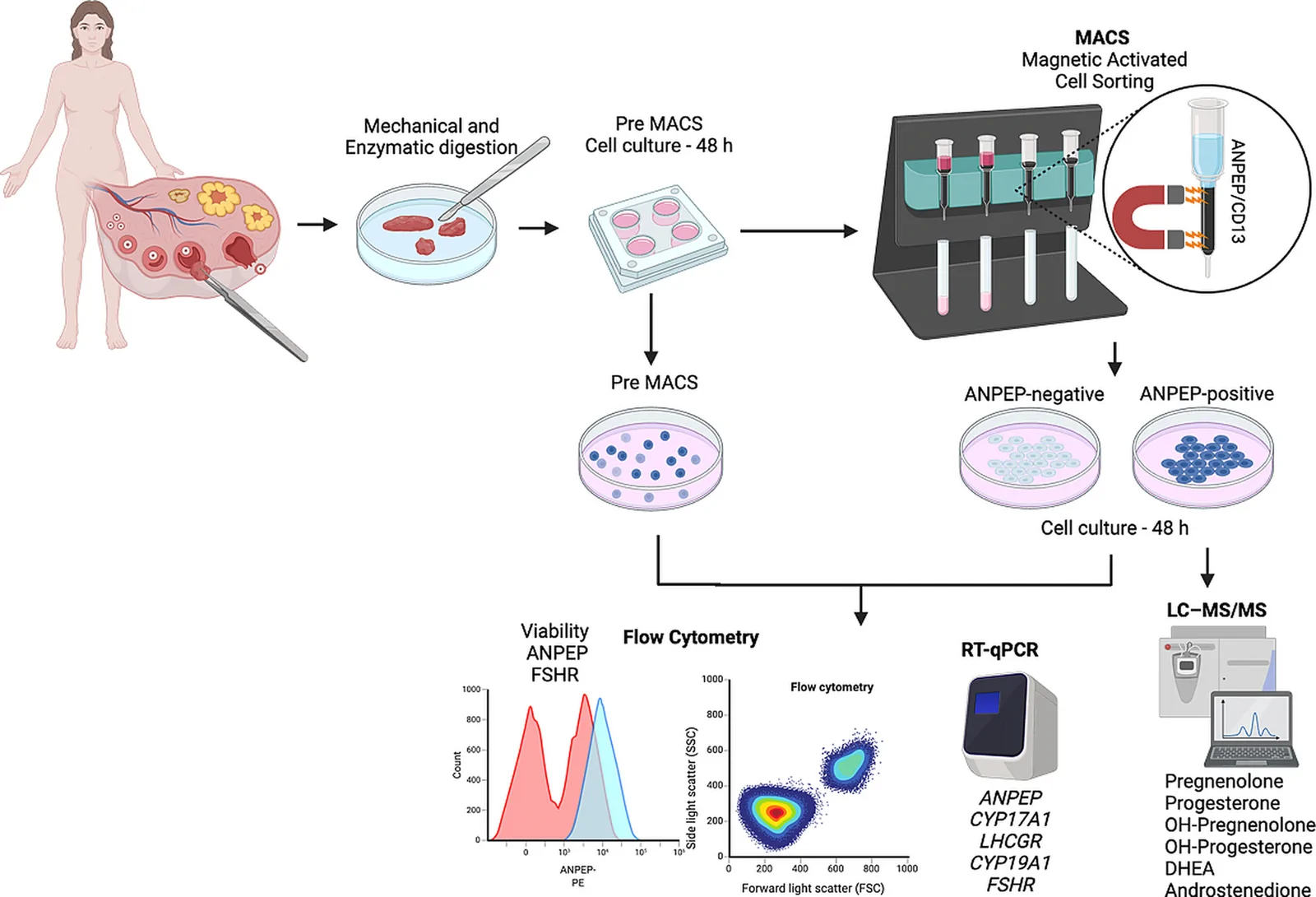
The experimental setup for the isolation of ANPEP-negative (granulosa) cells and ANPEP-positive (theca) cell populations from small antral follicle membranes collected from human ovaries of patients undergoing ovarian tissue cryopreservation, *n* = 8

Human theca cells were isolated from small antral follicle membranes following enzymatic digestion and magnetic sorting (Fig. 2). Three media types (holding, digestion, and termination) were prepared the day prior and pre-equilibrated overnight at 37 °C. Enzymes for digestion were pre-weighted and stored at − 20 °C. On the day of cryopreservation, follicular membranes were collected and dissected under a stereomicroscope to isolate theca-enriched fragments. Tissue was minced and incubated in digestion medium at 37 °C for 30–40 min with gentle pipetting every 10 min, on a shaker. The resulting suspension was washed twice in termination medium and centrifuged at 400 × g. Cells were resuspended in culture media, counted, and seeded in fibronectin-coated plates. After 48 h, cells were detached using Accumax, collected, and washed. A small fraction was lysed for gene expression analysis. The cells were labelled with a PE-conjugated anti-ANPEP antibody (5–10 µL per 10^6^ cells in 120 µL separation buffer) and incubated in the dark for 15 min at room temperature. Cells were washed to remove unbound antibody, then incubated for 15 min with anti-PE magnetic microbeads (20 µL per 10^7^ cells). ANPEP-positive and ANPEP-negative cell populations were separated using MS columns in a MACS magnetic separator. ANPEP-negative (unlabeled) cells were collected in the flow-through, while ANPEP-positive cells were retained in the column and subsequently eluted by removing the column from the magnet and flushing with separation buffer using a plunger. Each fraction was centrifuged, resuspended in culture media, counted, and seeded with a density of 100,000 cells pr. well, for 48 h. After 48 h, the two culture fractions were stained for flow cytometry, and a small fraction was lysed for gene expression analysis, and the culture media were snap-frozen for hormone analysis using mass spectrometry.

### Flow cytometry and immunostaining

The experimental setup is shown in Fig. 2. For immunostaining of surface proteins and intracellular receptors, cells were first washed with PBS and HEPES, harvested, and incubated with 400 µL Accumax (6–7 min, 37 °C) to detach adherent cells. The suspension was transferred to 10 mL tubes, diluted with 3 × volume of suspension buffer (SB), centrifuged at 400 × g for 5 min, and resuspended in 1 mL culture medium. Cells were counted, and 1 × 10^5^ cells per condition were aliquoted into 1.5 mL Eppendorf tubes. After centrifugation (400 × g, 5 min, 4 °C), pellets were resuspended in 500 µL ice-cold SB. For viability assessment, cells were incubated with viability dye (1 µL per 1 × 10^5^ cells in 500 µL SB) for 5–10 min at 4 °C in the dark, washed with 500 µL SB, and centrifuged. Cells were then blocked with 100 µL Fc-blocking buffer (10% goat serum in PBS) for 20 min on ice, washed, and resuspended in 100 µL SB. Surface staining was performed by incubating with pre-diluted primary antibodies; ANPEP (0.5 µL) or FSHR (1.0 µL) per 1 × 10^5^ cells in 100 µL SB for 20 min at 4 °C in the dark. Cells were washed, centrifuged, and fixed in 200 µL 2% paraformaldehyde (PFA) on ice for 15–20 min. After a final wash in 500 µL SB, samples were resuspended in 250 µL SB and analyzed by flow cytometry. Compensation controls included microbeads for ANPEP and FSHR, viability beads for viability stain, and an unstained control. For the cells, FMO (Fluorescence Minus One) and unstained cells were analyzed. Flow cytometry was carried out on a FACS LSRFortessa 3 laser using FlowLogic™, and FlowJo™ Software for analyzing the data output. The data were first detected as a cell population, using side scatter area (SSC-A) and forward scatter area (FSC-A) to minimize analysis of cell debris. Using FSC-W (Width) and FSC-A, the duplicates were excluded, and single cells were analyzed further. Non-viable cells were excluded from the single cells with FSC-A and the viability marker Pacific Blue viability stain. Single viable cells were analyzed using antibodies with a fluorophore for targeting specific cell lines, see Supplementary Figs. 1 and 2.

### RNA isolation and gene expression analysis by RT-qPCR

Total RNA was extracted from the cell samples previously stored in Trizol at − 80 °C. Upon thawing, samples were vortexed briefly and incubated at room temperature to equilibrate. Phase separation was initiated by adding 1-bromo-3-chloropropane, followed by centrifugation at 12,000 xg. The upper aqueous phase containing RNA was carefully collected and purified using the RNeasy Mini Kit (Qiagen), which utilizes a silica membrane-based spin column. Ethanol and wash buffers were applied sequentially to remove residual impurities, and RNA was eluted in RNase-free water. Purified RNA was stored at − 80 °C until downstream applications. The quantity and purity of RNA were determined using a NanoDrop™ spectrophotometer (2000/2000c, ThermoFisher Scientific). Measurements were taken in duplicate, and RNA concentrations were normalized to 0.1 µg/µL. Undiluted RNA samples were also preserved at − 80 °C.

Complementary DNA (cDNA) was synthesized from 100 ng of RNA using the High-Capacity cDNA Reverse Transcription Kit (Applied Biosystems™, 4,368,814), following the manufacturer’s protocol. Reactions were assembled using EconoSpin™ All-in-One Mini Spin Columns (Epoc Life Science, #1920) and processed in an Arktik Thermal Cycler (ThermoScientific, N11467) with the following thermal profile: 25 °C for 10 min for primer annealing, 37 °C for 120 min for reverse transcription, 85 °C for 5 min for enzyme inactivation, and finally held at 4 °C before storage at − 20 °C.

Quantitative real-time PCR (RT-qPCR) was carried out using TaqMan Fast Advanced Master Mix (ThermoFisher, 4,444,557) and gene-specific TaqMan probes in MicroAmp™ Optical 96-Well Reaction Plates (Applied Biosystems, N8010560). Reactions were performed using a QuantStudio™ 3 Real-Time PCR System (ThermoFisher Scientific), with the following cycling conditions: initial incubation at 50 °C for 2 min and 95 °C for 20 s (1 cycle), followed by 40 cycles of 95 °C for 1 s and 60 °C for 20 s, and a final cooling step at 60 °C for 20 s.

The following genes were analyzed, using TaqMan probes: Cytochrome P450 Family 19 Subfamily A Member 1 (*CYP19A1*, Hs00903411_m1)**,** Cytochrome P450 Family 17 Subfamily A Member 1 (*CYP17A1*, Hs01124136_m1)**,** Follicle Stimulating Hormone Receptor (*FSHR*, Hs01019695_m1)**,** and Alanyl Aminopeptidase (*ANPEP/CD13*, Hs00174265_m1), Luteinizing Hormone/Choriogonadotropin Receptor (*LHCGR*, Hs00174885_m1) with Glyceraldehyde-3-phosphate Dehydrogenase (*GAPDH*, Hs02786624_g1) serving as the endogenous control. Gene expression data were processed and analyzed using QuantStudio™ Design & Analysis Software (v1.5.2), applying the comparative Ct (ΔΔCt) method for relative quantification.

### Steroid hormone extraction from cell culture media

Solid phase extraction (SPE) was used to extract steroid hormones from the cell culture media in accordance with Weisser et al. [25]. In brief, SPE columns (500 mg Bond elute C18 cartridges with 3 mL reservoir, Agilent, USA) were placed in a vacuum manifold (IST Vacmaster, Biotage). Columns were pre-conditioned with 3 mL of MeOH and washed twice with 3 mL of LC-graded H_2_O (1 mL/min). The media samples were loaded onto the columns preloaded with 1 mL LC-graded H_2_O, and 50 µL internal standard (IS) in MeOH. An additional 2 mL LC-graded H_2_O was added, resulting in a total volume of 3 mL prior to extraction. The IS mix included deuterated analogues of key steroids at a concentration of 5 ng/ml: d_3_−17-OH-pregnenolone, d_3_−17-OH-progesterone, d_4_-pregnenolone, d_6_-DHEA, d_7_-androstenedione, and d_9_-progesterone. Enrichment proceeded at 1 mL/min. Columns were washed with twice 3 mL LC-graded H_2_O (3 mL/min) and 3 mL LC-graded H_2_O:MeOH (75:25, v/v). Analytes were eluted with 5 mL LC-graded H_2_O:MeOH (20:80, v/v, 1 mL/min), followed by evaporation to 500 µL under nitrogen at 60 °C. The elute were then transferred to LC vials, and the final volume was adjusted to 1 mL with LC-graded H_2_O:MeOH (90:10, v/v).

### Quantification of steroid hormones by liquid chromatography tandem mass spectrometry

Steroid hormones in the cell media were quantified using LC–MS/MS based on a fully validated method by Weisser et al. [25], with minor modifications. The method complies with the International Conference on Harmonisation (ICH) guideline (2005) [26].

Briefly, for online cleanup and chromatographic separation a binary 1290 Agilent Infinity LC system coupled with a 1100 Agilent HPLC pump was used. One hundred microliter sample was injected through a 0.3 µm in-line filter onto a C18 enrichment column (3.9 × 20 mm, 10 µm). Steroids were retained and impurities were washed to waste with an isocratic flow consisting of H_2_O:MeOH:formic acid (80:20:0.1, v/v/v) for 2 min at 1 mL/min. Steroids were separated using a gradient elution (mobile phase A: H_2_O + 0.1% formic acid; mobile phase B: 100% MeOH) increasing B from 10% to 99.5% over 14 min at 0.3 mL/min. Detection was performed using an AB SCIEX 4500 QTRAP mass spectrometer (APCI mode), with data processed in Analyst 1.6.2 software.

Chromatographic peaks from LC–MS/MS analysis were processed using MultiQuant 3.0 software from AB SCIEX. All chromatograms were reviewed and manually re-integrated when needed. Data were further processed in Microsoft Excel, and steroid concentrations were calculated based on ISs, with each steroid quantified relative to its corresponding deuterated analogue.

### Statistical analysis

Statistical analysis of steroid and gene expression data was performed using GraphPad Prism version 10.2.3 (GraphPad Software Inc., La Jolla, CA, USA). For comparison between paired theca and granulosa cell samples, a paired parametric t-test was applied when data were available from at least three biological replicates (n ≥ 3). The analyses were performed on matched samples obtained from the same patients. An unpaired Student’s t-test with Welch’s correction was applied for the statistical analysis of the gene expression to account for unequal variances between groups. Statistical significance was defined as *p* < 0.05, while *p*-values between 0.05 and 0.1 were considered indicative of a borderline trend.

Flow cytometry datasets were not subjected to statistical testing due to insufficient sample size. Comparisons were performed using ΔCt values normalized to *GAPDH*.

## Results

### Viability and specificity of enzymatically isolated cells from antral follicle membranes

To assess the viability and specificity of enzymatically isolated cells, FACS analysis was performed on the cell populations before MACS isolation.

**Fig. 3:**
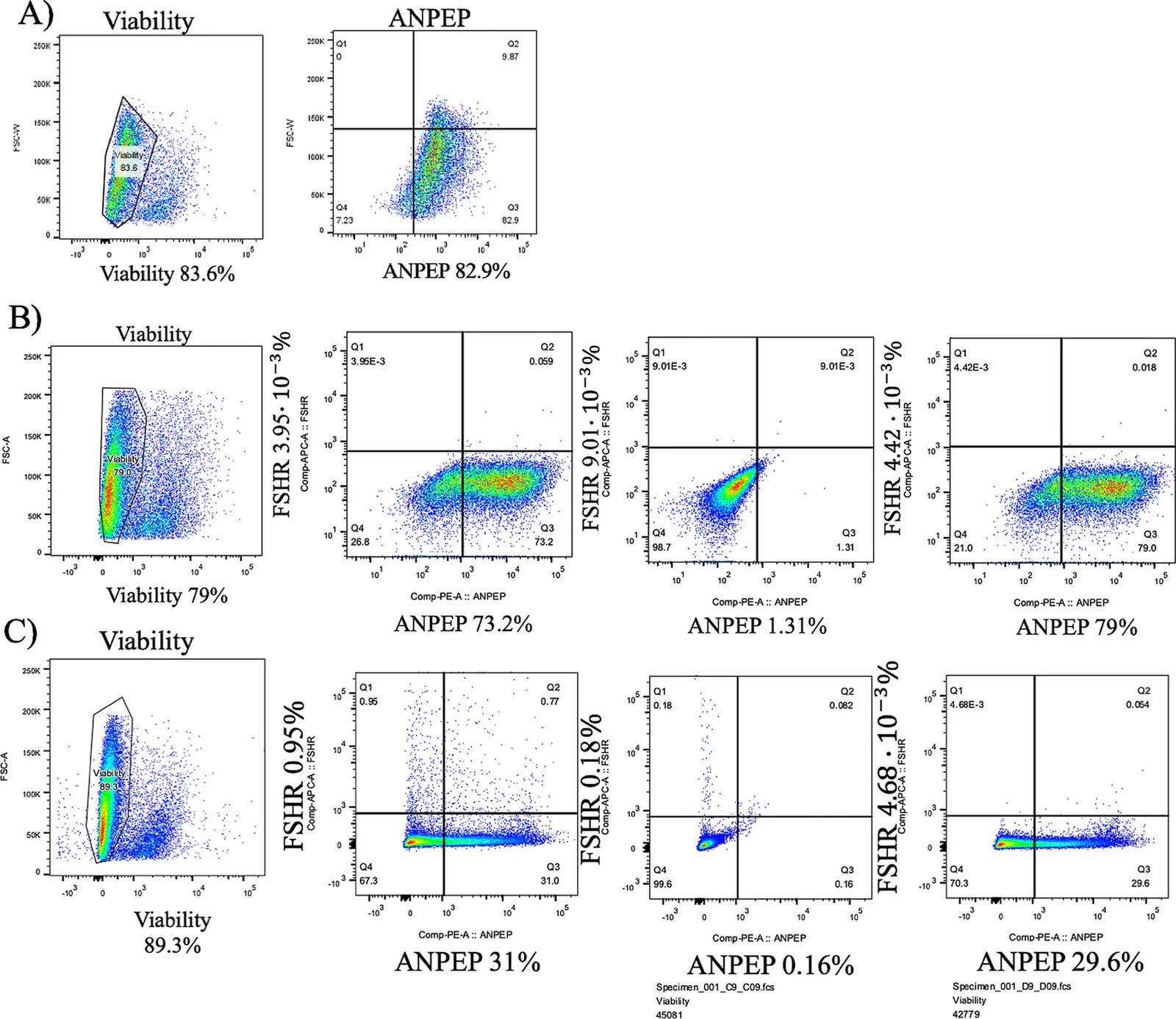
Flow cytometry analysis on mixed cell populations after 48 h of culture and pre-MACS, *n* = 8 patients. Three experiments, **A**, **B**, and **C**. **A** Cell viability and the number of viable cells expressing ANPEP on the cell surface. **B** and **C** Cell viability and ANPEP and FSHR expression on the cell surface

Pre-MACS isolation, and 48 h of culture after enzymatic digestion of the membranes retrieved from antral follicles, the culture combination consists of a range of 79% to 89.3% viable cells. Out of this population, a range going from 29.6% to 82.9% represents ANPEP on the membrane surface of theca cells. FSHR represents the membrane surface marker for granulosa cells in the population, which constitutes around 0.009% to 0.18%. However, the FMO for FSHR expressed, on average, 0.0046% in the population for FSHR, whereas the FMO for ANPEP, on average, expressed 0.74% of the cell surfaces (Fig. 3).

### ANPEP-based magnetic sorting enriches theca cell population from small antral follicles

**Fig. 4:**
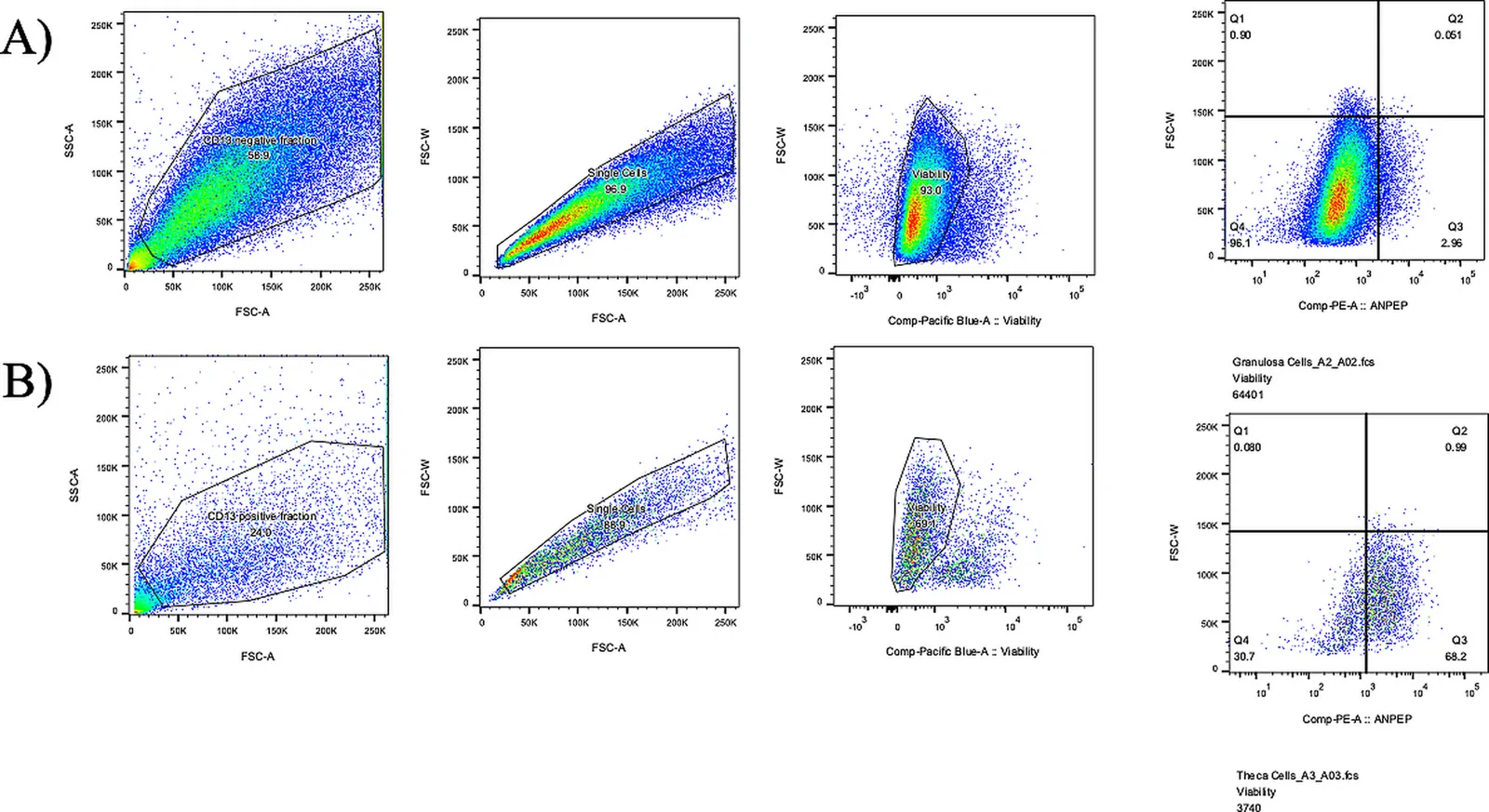
Flow cytometry, performed on the cell population after 48 h of culture and after MACS, shows cell viability and (**A**) unlabelled and ANPEP-negative, granulosa cell fraction. **B** ANPEP-positive, theca cells

For the samples analyzed using flow cytometry after MACS and 48 h of culture, the ANPEP-negative fraction was analyzed and consisted of 93%, and a total of 64,401 viable cells out of 100,000 analyzed cells. Only 2.96% of this fraction represented ANPEP. In the ANPEP-positive fraction, 69.1% were viable (*n* = 3740 cells), of which 68.2% expressed ANPEP on their surface, consistent with theca cell phenotype (Fig. 4).

### Sex steroid secretion during culture of ANPEP-negative and positive cells

To assess the functionality and steroidogenic capacity of magnetic sorted cell fractions, mass spectrometry analysis was performed on spent media from cell fractions cultured for 48 h after MACS. Steroid production was assessed in theca and granulosa cells across three independent experiments, including a mix of follicular membranes from different patients (Exp. 1–3), revealing distinct cell type-specific steroidogenic profiles.

In Experiment 1, pregnenolone was detected in both cell types, with slightly higher levels in the ANPEP-negative (granulosa) cells compared to the ANPEP-positive (theca) cells. Downstream metabolites in the Δ5 pathway showed clear differences, as 17-OH-pregnenolone and DHEA levels were markedly higher in theca cells than in granulosa cells. Similarly, androstenedione was detected at substantially higher levels in theca cells, whereas only low levels were observed in granulosa cells.

In Experiments 2 and 3, overall steroid levels were lower in both cell types, and differences between theca and granulosa cells were less pronounced, with several metabolites detected at low or near undetectable levels.

**Fig. 5:**
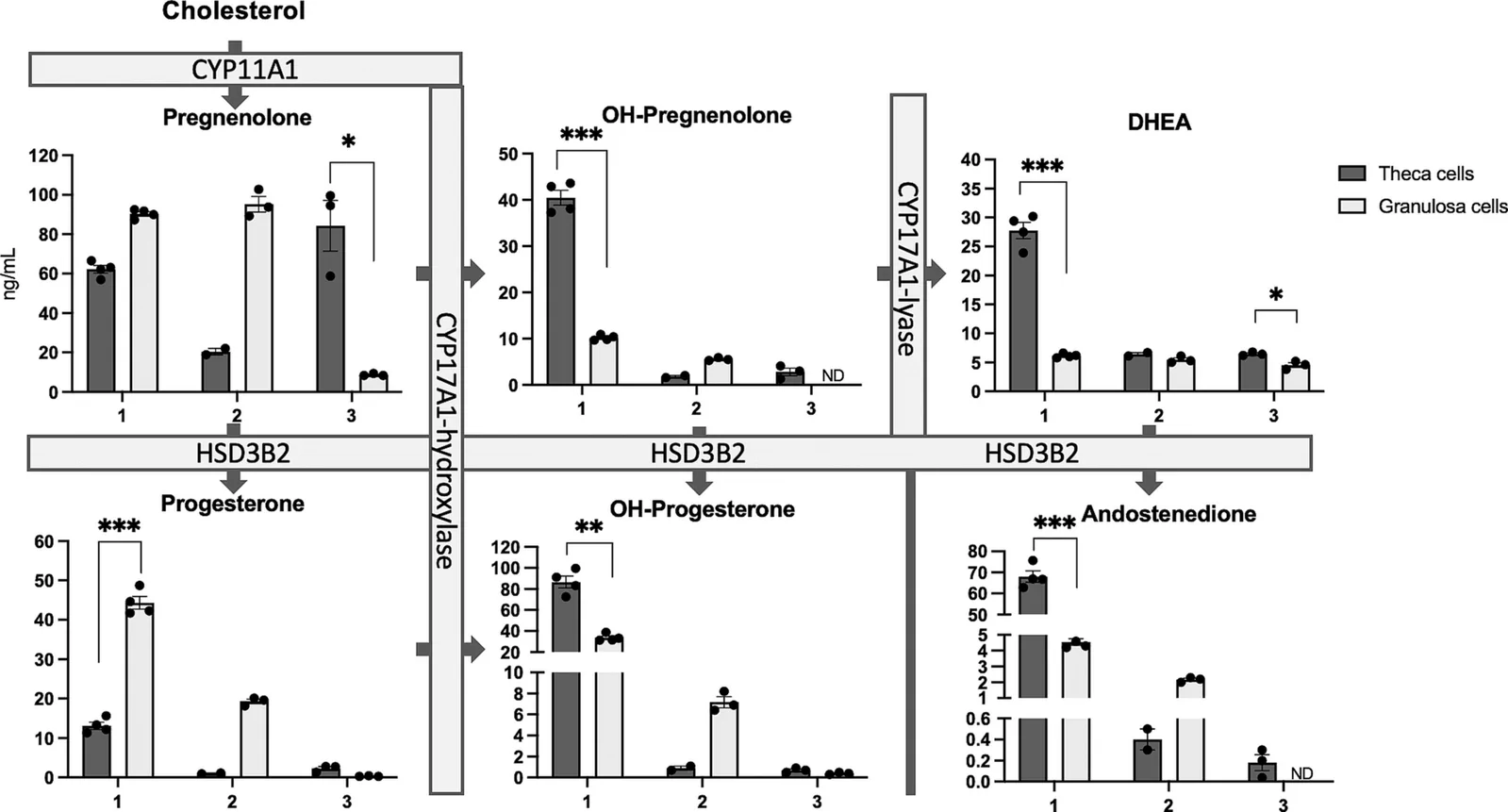
LC–MS analysis of spent culture media from three independent cell culture experiments following MACS isolation of ANPEP-positive (theca) and ANPEP-negative (granulosa) cells. The ovarian steroidogenesis pathway is illustrated with arrows, and the associated enzymes mediating each conversion are indicated. The *y*-axis represents metabolite concentration (ng/mL), and the *x*-axis denotes the three independent experiments (1–3). ND indicates non-detectable levels. No statistically significant differences were observed (*p* < 0.05). Error bars represent ± SEM (*n* = 8)

Three experimental setups were performed, each including two to three patients with biological replicates. Cells were seeded with a density of 100,000 cells pr. well. Steroid analysis of culture media included pregnenolone, progesterone, 17-OH-pregnenolone, 17-OH-progesterone, DHEA, and androstenedione. The ANPEP-positive fraction, representing theca cells (Fig. 5), secreted highly variable amounts of steroids across the three experiments. However, in all experiments, theca cells produced all six measured steroids, with higher overall levels observed in experiment one compared to the other two. The ANPEP-negative fraction, representing granulosa and stromal cells (Fig. 5), secreted comparable amounts of pregnenolone to the ANPEP-positive fraction (ANPEP-positive: 56 ng/ml ± 19 ng/ml; ANPEP-negative: 65 ng/ml ± 28 ng/ml), consistent with pregnenolone being an early steroid precursor produced by multiple ovarian cell types. In contrast, the ANPEP-negative fraction produced approximately two- to sixfold higher levels of progesterone than the ANPEP-positive fraction (21 ng/ml ± 13 ng/ml vs. 5.5 ng/ml ± 3.8 ng/ml, respectively). 17-OH-pregnenolone, 17-OH-progesterone, DHEA, and androstenedione showed higher secretion in the theca cell fraction compared to the granulosa cell fraction, as expected given the theca cells’ androgen precursor production. Steroid concentrations in theca and granulosa cell fractions are listed in Supplementary Table 1, and statistical significance is listed in Supplementary Table 2.

### Enrichment of theca-associated gene expression following MACS

**Fig. 6:**
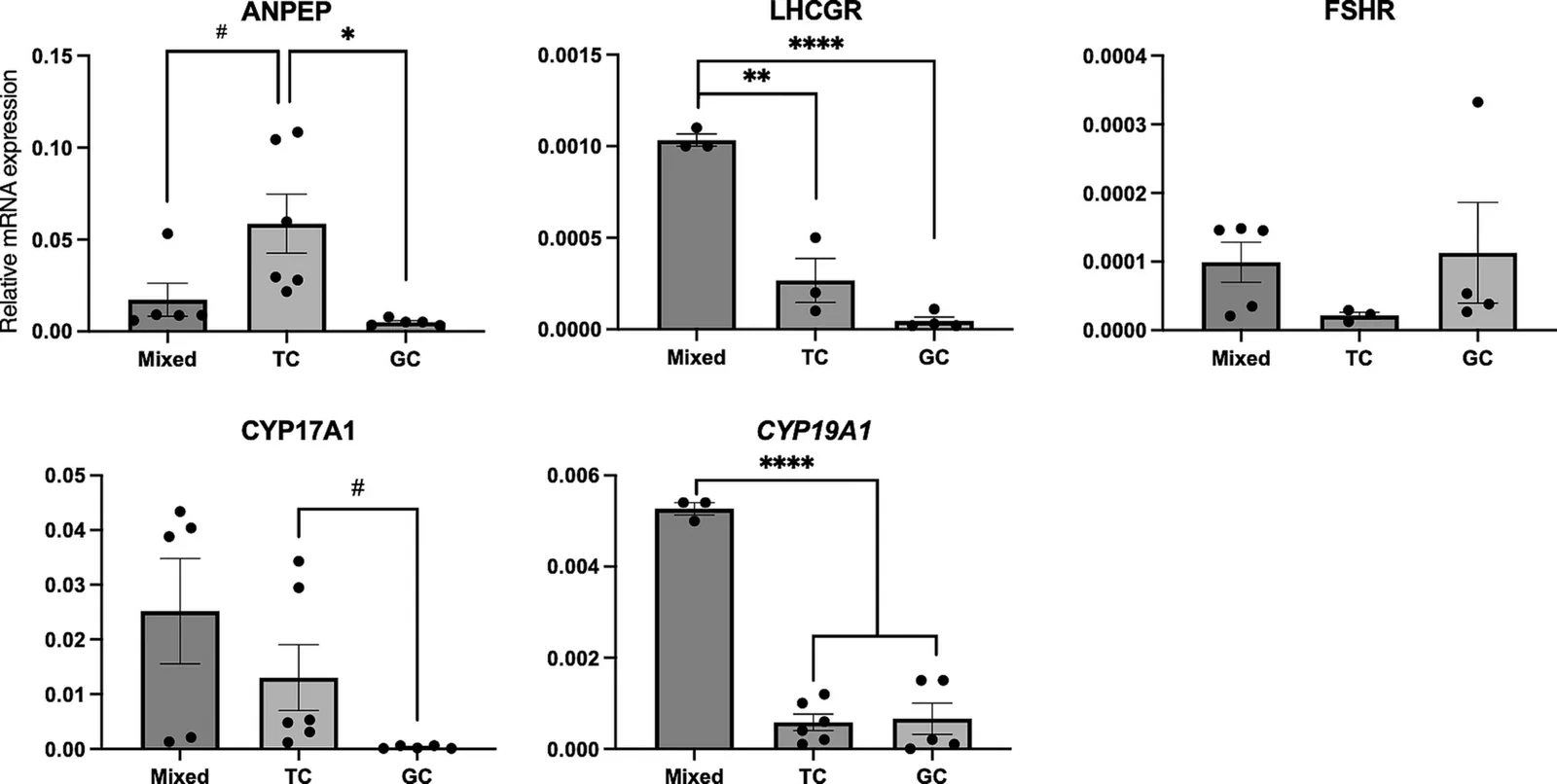
Gene expression analysis of cells in the mixed population prior to MACS isolation and after separation into ANPEP-positive (theca; TC) and ANPEP-negative (granulosa; GC) cells. The *y*-axis shows relative mRNA expression normalized to the housekeeping gene *GAPDH*. Error bars represent ± SEM (*n* = 8). Statistical significance was assessed using an unpaired *t*-test with Welch’s correction. Significance thresholds: #*p* = 0.05–0.1 (trend); *p < 0.05; **p < 0.01; ****p < 0.001

qPCR analysis was performed on cultured mixed cell populations pre-MACS, the ANPEP-positive fraction (theca cells), and the ANPEP-negative fraction (granulosa and stromal cells) (Fig. 6). The expression of *CYP17A1* and *ANPEP* was used as theca cell markers, while *CYP19A1* served as a granulosa cell marker. *ANPEP* expression was enriched in theca cells compared to granulosa cells (*p* < 0.05), with a borderline difference relative to mixed cells (*p* = 0.0557), while expression in granulosa cells was minimal. Similarly, *CYP17A1* expression was detected in mixed cells and theca cells but was negligible in granulosa cells, with borderline differences between theca and granulosa cells (*p* = 0.0887).

In contrast, *CYP19A1* expression was markedly higher in mixed cells compared to theca and granulosa cells (*p* < 0.0001), whereas expression in the sorted fractions remained low.

*LHCGR* expression followed the same pattern, with the highest levels observed in mixed cells and significantly lower in theca and granulosa cells (*p* < 0.005 and *p* < 0.0001, respectively).

*FSHR* expression did not differ significantly between groups, although expression levels tended to be lower in theca cells compared to mixed and granulosa cells.

Figure 6 shows that a significant difference was observed only in *ANPEP* and *CYP19A1* expression among the three populations. Specifically, *ANPEP* expression was higher in the theca cell fraction compared to the granulosa cell fraction (0.0049 ± 0.00082;* p* = 0.0204). *ANPEP* expression in the mixed population (0.017 ± 0.009) did not differ significantly from the granulosa cell fraction but showed a borderline trend toward lower expression compared to the theca cell fraction (*p* = 0.056).

*CYP17A1* expression was higher in both the mixed population (0.025 ± 0.0096) and theca cell fraction (0.0130 ± 0.006) compared to the granulosa cell fraction (0.0003 ± 0.0001); however, these differences were not statistically significant, although a borderline trend was observed between the theca and the granulosa cell fraction (*p* = 0.0887). Detailed gene expression and statistical analyses are provided in Supplementary Tables 3 and 4, respectively.

In contrast, *CYP19A1* expression was higher in the mixed population (0.004 ± 0.013) compared to the theca (0.00058 ± 0.00018) and granulosa cell fraction (0.00066 ± 0.00034), with statistically significant differences between the mixed population and both fractions (*p* < 0.0001), while no significant difference was found between the theca and granulosa cell fractions.

These findings support partial separation of theca and granulosa cell fractions at the transcriptional level. Detailed gene expression and statistical analyses are provided in Supplementary Tables 3 and 4, respectively.

## Discussion

### ANPEP-based MACS enables enrichment but not complete isolation of human theca cells

This study demonstrates that ANPEP-based MACS is a reliable alternative to FACS for enriching human theca interna cells. FACS showed a higher proportion of ANPEP-positive cells in the MACS sorted fraction, consistent with higher *ANPEP* gene expression levels observed in this fraction compared to the negative fraction. Together, these findings indicate that MACS enables effective, though only partial, purification of theca-lineage cells.

Consistent with this, expression of theca-associated marker *CYP17A1* was detected in both the ANPEP-positive fraction and in the mixed population, while remaining negligible in the ANPEP-negative fraction, supporting enrichment of steroidogenic active theca cells. Based on the two-cell, two-gonadotropin model of ovarian steroidogenesis, granulosa cell markers such as CYP19A1 and FSHR would be expected to be enriched in the ANPEP-negative fraction. However, this pattern was not observed: *CYP19A1* expression was highest in the mixed population and remained low in both sorted fractions, suggesting additional technical influences.

### Altered marker expression reflects limited granulosa cell representation and culture-induced changes

Granulosa cells from antral follicles are expected to express *FSHR* on their cell surface [27]; however, only a small proportion of FSHR-positive cells (0.18%) was detected in the mixed population before MACS. This may indicate a limited representation of granulosa cells in the starting material, but it could also reflect reduced *FSHR* expression under in vitro culture conditions or technical factors related to antibody sensitivity or epitope accessibility.

A plausible explanation is that granulosa cells underwent phenotypic changes during the 48-h culture period prior to MACS separation. It is well documented that granulosa cells luteinize in vitro, leading to downregulation of FSHR and altered steroidogenic gene expression, including reduced *CYP19A1* expression, and a shift toward increased *LHCGR* expression [28–31]. Thus, the lack of enrichment of CYP19A1 and FSHR in the ANPEP-negative fraction is likely to reflect a combination of limited granulosa cell abundance and culture-induced phenotype changes, rather than a complete absence of these cells.

Although *LHCGR* is expressed in both theca and granulosa cells, its regulation differs between these cell types, being constitutively expressed in theca cells but induced in granulosa cells during later stages of follicular development and luteinization. The elevated expression observed in the mixed population may therefore reflect contributions from both cell types, including granulosa cells at different stages of differentiation. Following MACS, the reduced *LHCGR* signal is therefore best explained by depletion of *LHCGR*-high granulosa cells, rather than reduced viability or marker expression.

### Sample variability and technical limitations in membrane isolation and MACS efficiency

A high percentage of ANPEP-positive cells prior to sorting (30%–83%) is consistent with the expected enrichment of theca cells in membranes derived from antral follicles. However, the substantial inter-sample variability likely reflects technical challenges associated with membrane isolation. In practice, precise identification and dissection of follicular membranes is difficult, and variable co-isolation of surrounding stromal and medullary tissue likely contributed to differences in the starting material. This heterogeneity in tissue composition likely contributes to the observed differences in ANPEP-positive cell proportions across samples.

Following MACS, the ANPEP-positive fraction contained approximately 68% ANPEP-positive cells, whereas only around 3% of cells in the ANPEP-negative fraction were ANPEP-positive, indicating effective enrichment but incomplete purification of theca cells. The incomplete recovery of ANPEP-positive cells may reflect limitations in capture efficiency during MACS, including suboptimal antibody labeling.

### Functional validation through steroidogenesis

Demonstration of steroidogenic activity is essential to confirm that phenotypically enriched cells have functional characteristics of the theca interna lineage. To complement the FACS validation of ANPEP-based separation, we assessed the steroid hormone secretion in culture media collected from the mixed population and the two sorted cell fractions. The well-established two-cell, two-gonadotropin model of ovarian steroidogenesis states that theca interna cells express *CYP17A1* and primarily produce androgen precursors, whereas the granulosa cells lack CYP17A1 and synthesize progesterone as a terminal product, while also aromatizing androgens from theca cells into estrogens under FSH stimulation [4, 32, 33].

In our study, the ANPEP-positive fraction consistently secreted pregnenolone, 17-OH-pregnenolone, 17-OH-progesterone, progesterone, DHEA, and androstenedione across the three experimental setups. Notably, several of these steroids, including 17-OH-pregnenolone, 17-OH-progesterone, and DHEA, are products of CYP17A1 activity and are characteristic of steroidogenic theca cells. In contrast, the ANPEP-negative fraction exhibited higher progesterone secretion with lower levels of androgen precursors, consistent with a granulosa cell population lacking CYP17A1 expression. These findings indicate that theca cells are the primary source of androgenic steroids, whereas granulosa cells predominantly produce progesterone, although this distinction was most evident in Experiment 1. The observed steroid profiles in the two fractions, therefore, align with canonical ovarian steroidogenic pathways and provide support for theca interna cells in the ANPEP-positive fraction. Steroid concentrations varied between experiments, with another study demonstrating up to an 82-fold difference in secretion between groups [29], likely attributable to variation in follicular stages and patient-specific effects. The alignment of ANPEP surface expression with enhanced androgen precursor production further indicates that the isolated ANPEP-positive fraction retains key steroidogenic characteristics of theca interna cells, supporting the biological validity of the MACS-based purification strategy.

### Limitations of MACS enrichment and sample variability

This study is limited by the sample size due to restricted access to human ovarian tissue from unstimulated patients undergoing OTC. In addition, the ANPEP-positive fraction reached approximately 68% after MACS separation, underscoring that this method enriches rather than fully purifies human theca cells, and here some heterogeneity is unavoidable. Accordingly, while ANPEP-based MACS represents a feasible and relatively simple enrichment strategy, the present findings do not provide sufficient evidence to conclude that it enables robust bulk isolation of authentic human theca cells with the precision required for downstream applications. Thus, the current approach should be regarded as a proof-of-concept enrichment method rather than a definitive isolation strategy, and further optimization is needed before it can reliably support mechanistic studies or broader research applications. Further purification by sequential MACS sorting was not feasible, as cells isolated using ANPEP-targeting magnetic beads would require an additional culture period to internalize and clear the initial bead–antibody complexes before a second magnetic labeling step could be performed. Such prolonged culture may alter cellular gene expression and phenotype, thereby introducing an additional confounding factor.

### Effects of *in vitro* culture and marker validation constraints

The 48-h culture period prior to sorting may have influenced marker expression, as primary ovarian cells can undergo phenotypic changes in vitro [29]. Although gene expression analysis revealed differences in steroidogenic markers, preliminary FACS experiments targeting CYP17A1 and CYP19A1 were performed; however, these experiments could not be sufficiently optimized due to limited cell numbers and technical challenges. Consequently, protein-level validation of these findings was not achieved. An additional limitation is that the isolation procedure required a 48-h culture period before cell sorting. Although this step was necessary to obtain sufficient cell numbers for downstream analyses, prolonged culture and enzymatic dissociation may influence gene expression and cell-surface marker profiles. Therefore, culture-induced effects cannot be fully excluded when interpreting the observed molecular signatures.

#### Implications of theca cell enrichment for modelling and clinical applications

Despite these limitations, enrichment of functional theca cells is important for modelling the two-cell steroidogenic axis. ANPEP-based MACS isolation provides a practical approach for obtaining theca-enriched populations for in vitro follicle models and studies of ovarian steroidogenesis. Such models enable a more accurate representation of the endocrine interplay required for follicle maturation and may be particularly relevant for investigating dysregulated androgen production, such as in polycystic ovary syndrome (PCOS) [34, 35]. Furthermore, access to unstimulated human follicles through OTC supports the development of regenerative ovarian constructs and hormone replacement strategies [36, 37]. In this context, functionally enriched theca cell populations hold significant clinical potential, both for use in bioengineered ovarian systems and long-term hormone replacement approaches [36, 37], as well as serving as a diagnostic model for theca cell-related disorders.

## Conclusion

This study demonstrates that MACS can be used to enrich theca interna cells from follicular membranes of unstimulated human ovaries. Although the resulting cell population is not fully homogeneous, the degree of enrichment achieved is sufficient to support the establishment of primary human theca cell cultures. Furthermore, this method does not appear to be suitable for isolating granulosa cells from the membranes of antral follicles, as a substantial loss of granulosa cells was observed after MACS sorting.

Overall, this provides a practical approach for obtaining theca-enriched populations from limited human follicular membranes. The ability to generate primary theca cell cultures from unstimulated ovaries enables the study of human ovarian function under more physiologically relevant conditions. Such models may facilitate investigations of theca cell function, steroidogenesis, and the cellular interactions underlying follicular development. This approach provides a method for future applications in reproductive biology and medicine, including disease modeling and the development of advanced in vitro systems to study ovarian function.

## Supplementary Information

Below is the link to the electronic supplementary material.

ESM 1 (PDF 3.84 MB)

ESM 2 (DOCX 26.4 KB)

## Data Availability

Some or all datasets generated during and/or analyzed during the current study are not publicly available but are available from the corresponding author on reasonable request.
