# Supplementary material for: Isolation of theca cells from human antral follicle membranes using ANPEP-based magnetic cell sorting
Source: J Assist Reprod Genet. 2026 Jul 23;43(9):3007–19. doi: 10.1007/s10815-026-03983-2 (PMC13731582; doi:10.1007/s10815-026-03983-2)
Supplement: Supplementary file 1 [file 10815_2026_3983_MOESM1_ESM.pdf]

Supplementary Figure 1: Pre-MACS isolation of cells

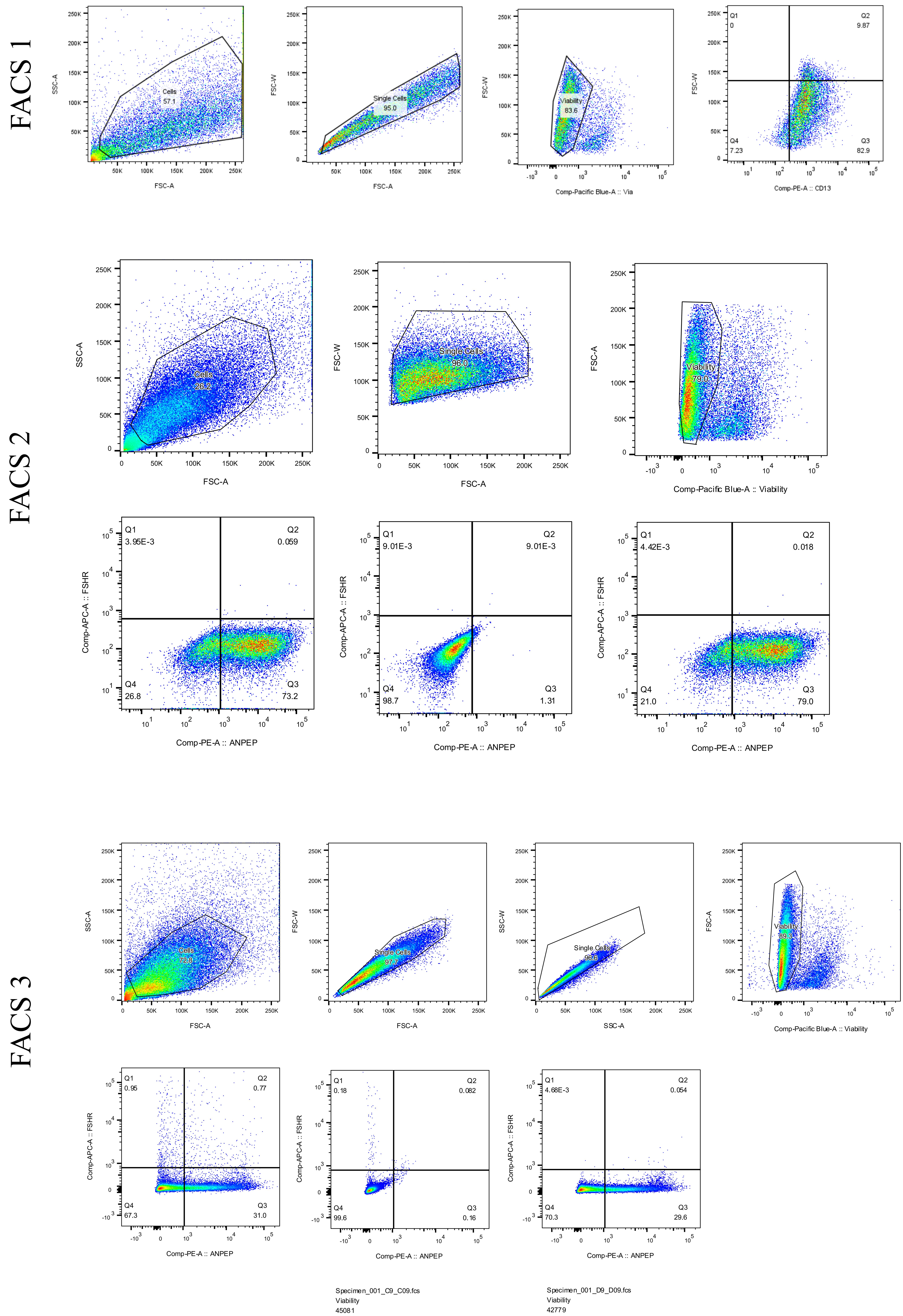

Supplementary Figure 2: Post MACS isolation of cells – ANPEP-positive and ANPEP-negative

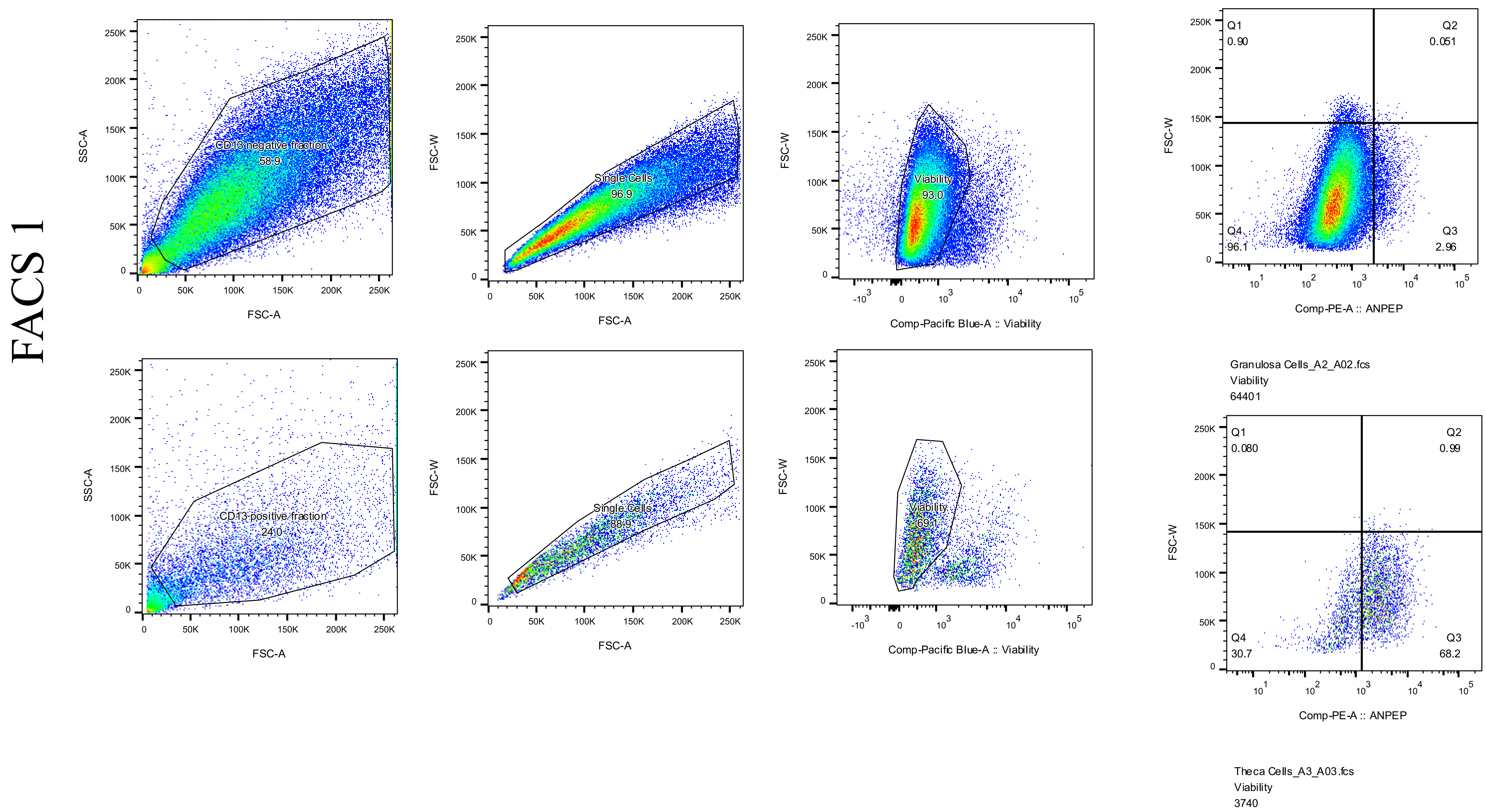

Supplementary Fig 1 Flow cytometry analysis on mixed cell populations after 48 hours of culture and before MACS. FACS 1, FACS 2, and FACS 3 show three different cell cultures. FACS 1 demonstrates cell viability and the number of viable cells expressing ANPEP on the cell surface. FACS 2 and FACS 3 demonstrate cell viability and ANPEP and FSHR expression on the cell surface

Supplementary Fig 2 Flow cytometry, FACS 1, performed on the cell population after 48 hours of culture and after MACS. Shows cell viability, and the right upper corner is unlabelled, ANPEP-negative, granulosa cell fraction. The bottom right-hand corner shows ANPEP-positive, theca cell fraction
