# Supplementary material for: Isolation of theca cells from human antral follicle membranes using ANPEP-based magnetic cell sorting
Source: J Assist Reprod Genet. 2026 Jul 23;43(9):3007–19. doi: 10.1007/s10815-026-03983-2 (PMC13731582; doi:10.1007/s10815-026-03983-2)
Supplement: Supplementary file 2 [file 10815_2026_3983_MOESM2_ESM.docx]

**Supplementary Table 1** Steroid concentrations in theca and granulosa cells

|  |  | Theca cells | | | Granulosa cells | | |
| --- | --- | --- | --- | --- | --- | --- | --- |
|  |  | Exp. 1 | Exp. 2 | Exp. 3 | Exp. 1 | Exp. 2 | Exp. 3 |
|  |  | ng/mL | | | | | |
| Pregnenolone | Mean ± SEM | 62 ± 2 | 20 ± 1.6 | 84 ± 13 | 90 ± 1.2 | 95 ± 4.0 | 8.6 ± 0.3 |
| Progesterone | Mean ± SEM | 13 ± 0.93 | 1.0 ± 0.10 | 2.3 ± 0.5 | 44 ± 1.6 | 19 ± 0.57 | 0.3 ± 0.03 |
| 17-OH-pregnenolone | Mean ± SEM | 40 ± 1.6 | 1.8 ± 0.2 | 2.8 ± 0.8 | 10 ± 0.3 | 5.6 ± 0.2 | ND |
| 17-OH-progesterone | Mean ± SEM | 87 ± 5.8 | 0.9 ± 0.2 | 0.7 ± 0.1 | 34 ± 1.7 | 7.2 ± 0.5 | 0.4 ± 0.06 |
| DHEA | Mean ± SEM | 28 ± 1.4 | 6.4 ± 0.3 | 6.5 ± 0.2 | 6.2 ± 0.2 | 5.5 ± 0.3 | 4.5 ± 0.4 |
| Androstenedione | Mean ± SEM | 68 ± 2.7 | 0.4 ± 0.1 | 0.2 ± 0.08 | 4.6 ± 0.2 | 2.2 ± 0.09 | ND |

**Supplemental Table 2** Steroid concentrations in paired theca and granulosa cell samples.

| Steroid | Patient | *p*-value | Statistical significance |
| --- | --- | --- | --- |
| Pregnenolone | 3 | 0.0270 | * |
| Progesterone | 1 | 0.0005 | *** |
| OH-Pregnenolone | 1 | 0.0002 | *** |
| OH-Progesterone | 1 | 0.0029 | ** |
| DHEA | 1 | 0.0008 | * |
| DHEA | 3 | 0.0151 | * |
| Androstenedione | 1 | 0.0002 | *** |

**Supplementary Table 3** ΔΔCt-values for ANPEP, CYP17A1, and CYP19A1 in mixed cells, theca cells, and granulosa cells

|  |  | ANPEP | CYP17A1 | CYP19A1 |
| --- | --- | --- | --- | --- |
|  |  | ΔΔCt-values | | |
| Mixed cells | Mean ± SEM | 0.017 ± 0.009 | 0.025 ± 0.0096 | 0.004 ± 0.0013 |
| Theca cells | Mean ± SEM | 0.059 ± 0.016 | 0.013 ± 0.006 | 0.00058 ± 0.00018 |
| Granulosa cells | Mean ± SEM | 0,0049 ± | 0.00032 ± 0.00012 | 0.00066 ± 0.00034 |

**Supplementary Table 4** Statistical significance.

|  |  | Granulosa cells vs mixed | Theca cells vs mixed | Theca cells vs granulosa cells |
| --- | --- | --- | --- | --- |
| ANPEP | p-value | ns | 0.0557^#^ | 0.0204 (<0.05) |
| CYP17A1 | p-value | ns | ns | 0.0887^#^ |
| CYP19A1 | p-value | <0.0001 | <0.0001 | ns |

^#^ Borderline
